# Sustainable bacterial cellulose production by *Achromobacter* using mango peel waste

**DOI:** 10.1186/s12934-023-02031-3

**Published:** 2023-02-06

**Authors:** Mohamed S. Hasanin, Mohamed Abdelraof, Amr H. Hashem, Houssni El Saied

**Affiliations:** 1 Cellulose and Paper Department, National Research Centre, Cairo, 12622 Dokki Egypt; 2 Microbial Chemistry Department, National Research Centre, Cairo, 12622 Dokki Egypt; 3 Botany and Microbiology Department, Faculty of Science, Al-Azhar University, Nasr City, Cairo, 11884 Egypt

**Keywords:** Bacterial cellulose, Achromobacter, Agricultural wastes, Optimization

## Abstract

Bacterial Cellulose (BC) is still the most renewable available biopolymer produced in fine nature from alternative microbial sources as bacteria. In the present study, newly BC producing bacteria were successfully isolated from acidic fruits. The most potent producer was isolated from strawberry and identified genetically using 16 s rRNA technique as *Achromobacter* S3. Different fruit peels were screened to produce BC using the cheapest culture medium. Among them, Mango peel waste (MPW) hydrolysate proved to be the significant inducible alternative medium without any extra nutrients for the maximum productivity. Improvement of the BC yield was successfully achieved via statistical optimization of the MPW culture medium, from 0.52 g/L to 1.22 g/L with 2.5-fold increased about the standard HS culture medium. Additionally, the physicochemical analysis affirmed the cellulose molecular structure as well as observed the crystallinity of nanofiber as 72 and 79% for BC produced by *Achromobacter* S33 on HS and MPW media, respectively. Moreover, the topographical study illustrated that the BC nanofibers had close characteristics upon fiber dimeter and length as about 10 and 200 nm, respectively.

## Introduction

Cellulose is one of the most abundant polysaccharide on Earth produced by a large member of living organisms such as plant, microorganisms, and animals [1]. Although its production is mostly by higher plants, an alternative route by bacterial cells system was very preferred and desired due to its characterized as low-cost, rapid resource, and easily purification steps. Plant cellulose was usually extracted and purified from lignin, and hemicellulose that existence naturally in the plants, which was required many complicated purification steps [2, 3]. Bacterial cellulose (BC) is fine cellulose commonly produced by Gram-negative bacteria in several forms with unique characteristics, specifically used in the biomedical materials. Interestingly, an easily extraction and few purification steps of BC was make it a promising alternate to utilized instead of the plant cellulose in several fields [2]. Discovering of new BC producer with high production efficacy and development of novel culture medium using agricultural wastes to obtain a sustainable culture medium was become more desirable. In addition, finding a new BC network with novel characteristics can be highly appreciable for application in the biomedical fields. Therefore, isolation of a new BC producers from different wastes is considered as a potent direction to get a novel BC prosperities. The major BC producers such as, *Pseudomonas*, *Acetobacter,* and *Gluconacetobacter* was frequently reported. The most potent model strain *Gluconacetobacter xylinus* was extensively used for basic and applied studies on BC [4]. Formation of BC was usually conducted via static condition, which the bacterial strain was formed the cellulose network as pellicle over the culture surface [5–7]. Moreover, the ability of BC production using agricultural waste hydrolysates may be promoting a large scale production of BC. Otherwise, the synthetic culture medium which widely used in the BC production like Hestrin–Schramm HS [8] was related with the lower productivity and higher in cost [7]. Cost-effectiveness of the cultural medium is considered as an important factor in the production process economics [6]. Accordingly, finding a new culture medium that characterized as inexpensive in particular that rich in organic matter was become very desirable in order to meet the industrial sectors requirements [1]. Because the synthetic HS culture medium has different drawbacks that causing higher feedback inhibition in the BC productivity like pH value, in which the pH was rapid conversion toward acidic value. Due to the fast catabolism of glucose by bacterial cells and conversion to excess of gluconic acid which contributing in the lowered of pH value [7]. Different agricultural wastes were extensively investigated by many authors to solve these problems [9–11]. Currently, studies have interested on various bacterial cellulose producers, various agricultural wastes, and supplementary materials for the low-cost BC with unique chemical and physical properties [12]. Furthermore, exploitation of wastes derived from food industries could be related with great performance in the food industrial sector and developed many solutions on this side within the last decades. Previous studies reported that rotten fruits can be used for BC production [13, 14] in order to reduce industrial waste amount and find a suitable cultural medium for BC production. In this regard, agricultural waste materials and byproducts from food industrial locations have already been investigated as an alternative source to obtain the maximum BC yield and at the same time decrease the BC production cost [7]. For example, dry olive mill residue, sugarcane molasses, waste beer yeast, wastewater from candy processing, wood sugars, waste from fruit processing, lipid fermentation wastewater, rice bark, konjac powder, cotton-based textile waste, and coffee bean husks were explore as a culture medium for the BC production [15–21]. In fact, the utilization of further agricultural wastes was increased the sustainability of BC production as well as reducing the environmental contamination [9]. Therefore, the high exploitation of agricultural wastes is attributed with many beneficial in different sectors such as economics, environment and practicality [7]. In this way, a huge quantity of fruit peels produced from juice factories remains after industrial processing, which is mostly not suitable for consumed by non-ruminants without chemical treatments due to its fibrous contains like pomegranate, mango, pineapple, and citrus peels [1]. Those candidates it as a cheap material valid to be sustainable culture medium and available base material for fermentation processes. Therefore, the present study is aimed to isolate a new bacterial strain has ability to produced nanocellulose fibers with high productivity using alternative medium form fruit peel waste hydrolysate. The produced BC was characterized via physicochemical topographical studies. The productivity and dependent variables, Response Surface Methodology (RSM) was used with central composite design method for enhancing BC production.

## Materials and method

### Collection of samples

Six ripe fruit without disease symptoms were collected from disposal of free markets namely, Strawberry, Pineapple, Citrus orange, Apple, Pomegranate, and Tomato. The selected fruits were washed thoroughly with bi-distilled water to remove additional residues from its surface.

### Isolation of bacteria from fruits

Each of fruit was first mixed with sterilized bi-distilled water (0.1 g/ml w/v) and then grounded aseptically by a stomacher bag and shaking for 2 h. at 180 rpm. Serial dilutions (10^− 2^–10^− 6^) were performed, and dilutions were plated in duplicate on the isolation medium (g/L) (sucrose 5, beef extract 1.5, Na_2_HPO_4_ 0.44, citric acid 0.08, agar 20.0, and ethanol 1 mL/L, pH 6.0) [22]. Each individual distinct transparent gel granular colonies were isolated and repeatedly purified by repeated streaking onto new agar plates until single colony morphology was observed. The obtained single colonies were preserved on nutrient agar slants at 4 °C for screening studies.

### Screening of cellulose-producing bacterial isolates

All bacterial isolates were then screened for their ability to formation of BC pellicle in the standard HS culture medium surface by inoculating each in statically condition at 28 °C for 2 weeks. The screening medium (Standard HS medium) consisted of (w/v) 2% glucose, 0.5% peptone, 0.5% yeast extract, 0.27% disodium phosphate and 0.115% citric acid monohydrate [8]. Only the bacterial isolate that showed the pellicle formation in the surface of culture medium was selected as the potential cellulose-producing bacterial isolate for further studies**.** The resulting pellicles were harvested by centrifugation at 4000 g for 10 min at room temperature and rinsed with distilled water to remove residue medium components, attached bacterial cells and other impurities. The pellicles were then boiled in 0.1 M NaOH solution for 20 min in order to eliminate the attached bacterial cells [14, 23, 24]. The pellicles were washed thoroughly with bi-distilled water for 2–3 times. Finally, the purified pellicles were dried at 70 °C until a constant weight is obtained and weighed to determine the BC production (g/L) [1]. After comparing the BC productivity (g/L) of each potential isolates, bacterial isolate that produced maximum yield of BC was chosen as the most potent cellulose-producer and subjected to identification studies.

### Molecular identification of the most potent cellulose-producing isolate

The highest BC producer isolate was then subjected to identification process using molecular characterization via 16 s rRNA. In this regard, extraction of total genomic, amplification using PCR, purification, and sequencing of the targeted gene were carried out by Macrogene protocol (Seoul, South Korea; https://www.macrogen.com). Universal Forward primer 27f (5′-AGAG TTTGATCCTGGCTCAG-3′) and reverse primer 1492r (5′-GGTT ACCTTGTTACGACTT-3′), was used for the PCR-amplification of 16 s rRNA gene. PCR condition was justified as described by (Abdelraof et al., 2020), and the resulting amblicon was purified and then sequenced by the ABI 3730 DNA Analyzer (Applied Biosystems). Alignment of the resulted sequence and compared it with other sequences recorded in the Genebank was carried out using the Basic Local Alignment Search Tool (http://blast.ncbi.nlm.nih.gov/Blast.cgi). Phylogenetic tree was then constructed were aligned with the sequences of representative strains by Molecular Evolutionary Genetic Analysis Software (MEGA version X). Subsequently, the obtained sequence of 16 s rRNA gene of the most promising cellulose-producer isolate in this study was deposited in Genebank database under accession number MZ381282, and the strain was identified as *Achromobacter* S33.

### Screening of different fruit peels hydrolysate for BC production

Several fruit peels were screening for their ability to serve as sustainable production medium for the BC by *Achromobacter* S33. Peels of apple, citrus orange, banana, pomegranate, pineapple, and mango were collected and prepared its hydrolysate using nitric acid treatment as described by [1]. Screening of peel hydrolysates were conducted in comparison with the standard HS culture medium for 7 days under static condition at 28 °C. Selection of the preferred hydrolysate was carried out according to the cellulose productivity.

### Central composite design (CCD)

In order to investigate the relationship between independent and dependent variables, RSM was used with one of the central composite design method, a central composite face-centered (CCF) design [25, 26]. In this study, independent factors PH, inoculum size and mango peel with different five levels as follow PH 3, 4, 5, 6 and 7 & inoculum size 2, 3, 4, 5 and 6% & mango peel 20, 30, 40, 50 and 60% were used as shown in Table 1. CCD design (20 runs) was designed using factors and their levels as shown in Table 2. The significance of the model was determined by analysis of variance, the regression equation was obtained, a P value less than 0.05 indicates that the model term is significant.

**Table 1:** Factors and their levels affecting BC production

Factor	Level 1	Level 2	Level 3	Level 4	Level 5
pH	3	4	5	6	7
Inoculum size %	2	3	4	5	6
MPW %	20	30	40	50	60

*MPW* mango peel waste

**Table 2:** Isolation of BC from local acidic fruits

Isolation source	No. of isolates	No. of BC producer isolates	BC isolate code	BC yield (g/L)
Strawberry	14	4	S33	0.48 ± 0.02
			S4	0.22 ± 0.03
			S9	0.21 ± 0.08
			S13	0.32 ± 0.00
Pineapple	8	0	0	0
Citrus orange	6	0	0	0
Apple	11	1	M1	0.09 ± 0.01
Pomegranate	7	0	0	0
Tomato	13	3	T2	0.20 ± 0.03
			T4	0.17 ± 0.08
			T5	0.07 ± 0.02

### BC characterization

The physiochemical characterizations were studied via Attenuated total reflection Fourier-transform infrared (ATR-FTIR) spectroscopy “Spectrum Two IR SpectrometerPerkinElmer, Inc., Shelton, USA”. Spectral analysis was obtained at 32 scans and 4 cm^−1^ resolutions in wavenumbers ranging from 4000 to 400 cm^−1^. The X-ray (XRD) diffractometer, Schimadzu 7000, Japan, in the 2θ range from 20 to 70° in 0.02° steps at λ = 1.5418Å using CuKα radiation was used to study the crystallography. The crystallinity (Cr.I), the degree of samples was calculated according to the following equation (Eq. (1)) [27, 28]:

1 $$Cr.I = \left( {\frac{{I_{{200}} - I_{{am}} }}{{I_{{200}} }}} \right) \times 100$$

where I_200,_ corresponding to the crystalline, is the height of the peak intensity at lattice diffraction 002 and 2θ = 22.4°, while I_am_, corresponding to the amorphous fraction, is the height of the minimum peak intensity between 002 and the 101 peaks. The I_am_ value appeared almost around 2θ = 18°.

The topographical studies were cariied out via scanning electron microscopy (SEM, Quanta FEG 250, FEI, Republic of Czech) as well as high-resolution transmission electron microscope (HRTEM) JEOL–JEM-1011, Japan. The images analysis was carried out using ImageJ free software.

### Statistical analyses

The experimental results included in this study were expressed as the average ± standard deviation (SD) for n = 3 and were analyzed using standard analysis of Student’s *t*-test.

## Results and discussion

### Isolation and screening of cellulose-producing isolates

Among six acidic local fruits, a total of 59 bacterial isolates were picked up in order to evaluate its capability to formation of cellulose pellicle. Selection of acidic fruits as an isolate host in our study due to the cellulose-producing bacterial strains prefers acidic condition for growth [4]. Previous studies revealed that the optimum pH range for BC production is ranged from 4 to 7 [5]. In addition, most of the BC-producers were isolated from acidic plants such as apple, orange, tomato, grape and wild lemon [6].

**Fig. 1:**
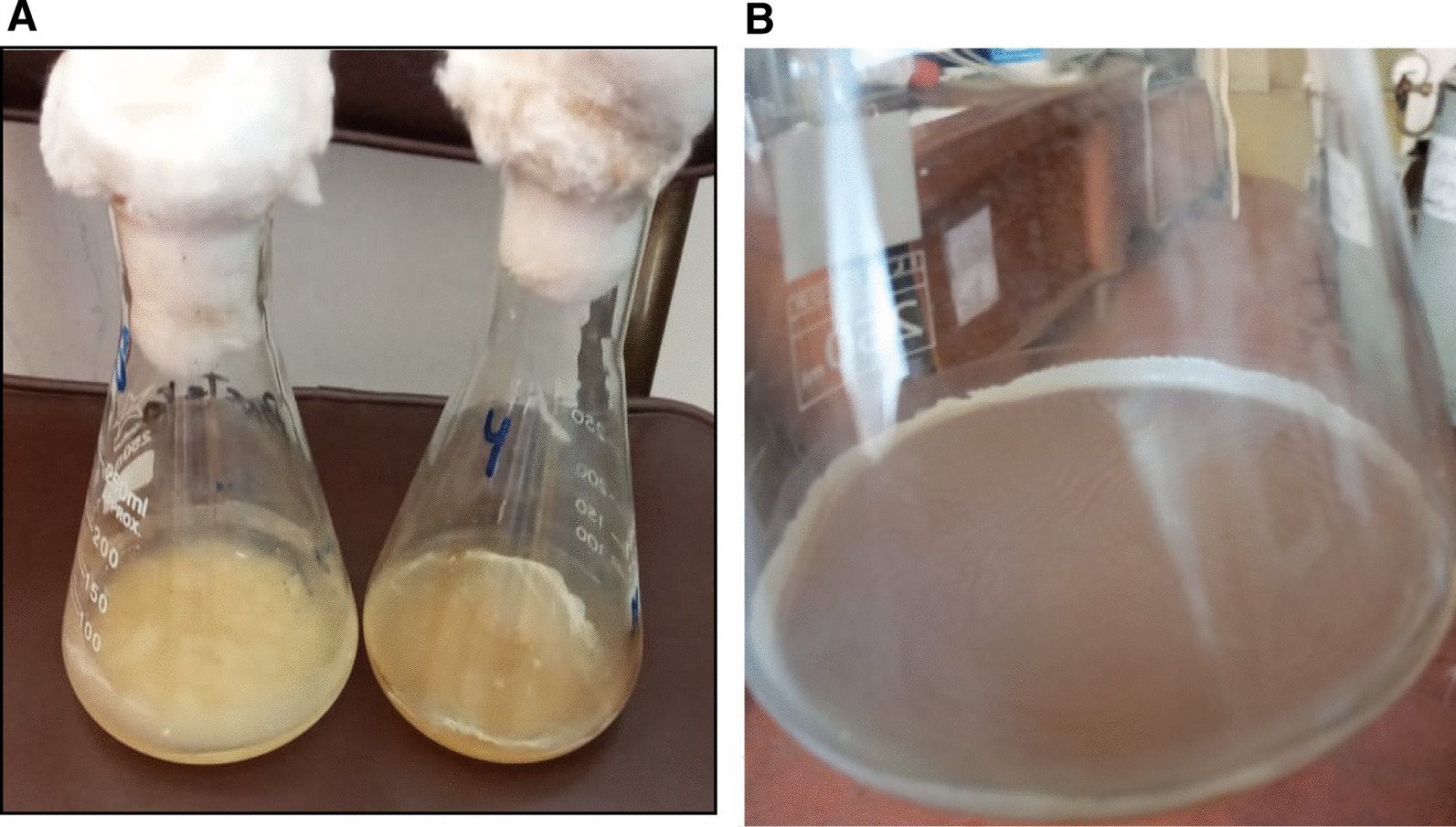
The most potent cellulose-producing isolates: **A** Isolate S4, and S13; **B** S33 grown on HS broth medium

Among six acidic local fruits, a total of 59 bacterial isolates were picked up in order to evaluate its capability to formation of cellulose pellicle. As shown in Table 2, eight bacterial isolates (approx. 13.5% of the total 59 bacterial isolates) were able to produce pellicle at the air–liquid interface of the culture medium. From the six local acidic fruits, we noted that strawberry fruit was the preferred source of cellulose-producing bacteria, which provided four potential bacterial isolates, approx. 6.77% of the total 59 bacterial isolates. Besides, the isolates from strawberry were provided thick film of cellulose pellicle than other producer isolates from tomato and apple. The results showed the isolate code as S33 (derived from strawberry fruit) demonstrated the maximum BC yield (0.48 g/L), followed by the isolate S13 produced the second highest BC productivity with 0.32 g/L (Fig. 1). Meanwhile, the lowest BC yield was obtained from the apple isolate (0.09 g/L).

In accordance of our results, most of BC-producers were isolated from fruits, particularly acidic ones [29]. Suwanposri, Yukphan, Yamada and Ochaikul [30] isolated cellulose-producing bacterium from the tropical fruits such as mangosteen, papaya, rambutan, and watermelon. Rotten apple was also used as a source of *Gluconacetobacter hansenii* isolation, which was produced about 0.35 g/L of BC [31]. *Gluconacetobacter xylinus* isolated from kombucha could be synthesize 0.28 g/L of BC by using HS medium after 7 days of cultivation period at 30 °C under static condition [32]. Indeed, the BC yield in the present study was satisfied as compared with the fruit isolates reported in the previous studies. Therefore, it is thought that the most potent bacterial isolate S33 could be identified as a newly isolated cellulose-producing bacteria and optimization of the culture conditions should be conducted in further study to improve the BC production yield.

### Bacterial identification by 16S rRNA gene sequence analysis

**Fig. 2:**
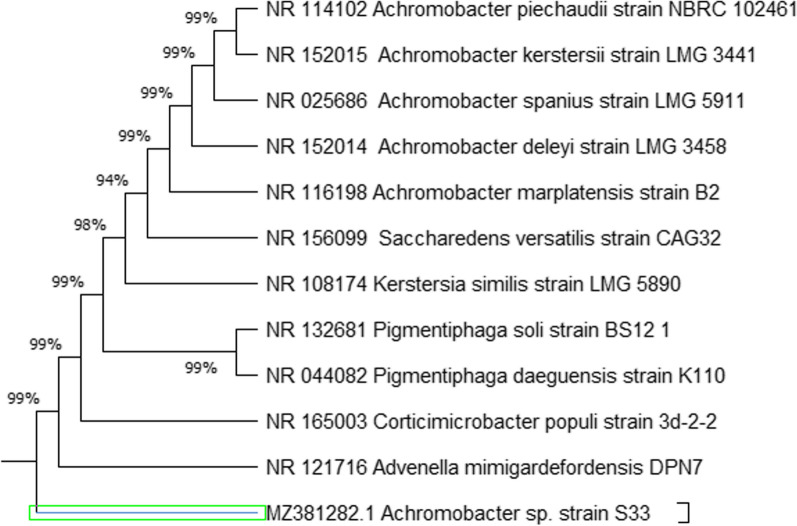
The phylogenetic tree of S3 isolate constructing by MEGA X

The identification of the most potent cellulose-producing isolate has been confirmed based on the molecular characterization. The widely molecular characterization used method of 16S rRNA gene analysis was applied; PCR amplification of 16S rRNA gene was conducted using universal primers. Results illustrated that the almost complete sequence of 16S rRNA gene had 99% similarity with *Achromobacte*r strain. 16S rRNA sequences from the *Achromobacter* species were obtained from the NCBI (www.ncbi.nlm.nih.gov/) and a phylogenetic tree was constructed by the neighbor-joining method (Fig. 2). Therefore, according to the morphological observations and molecular analyses, the results suggested that this isolate S33 was closely related to *Achromobacter* strain and designated as *Achromobacter* S33 (GenBank accession number: MZ381282). To the best of our knowledge, this is the first study that isolated *Achromobacter* Sp. from strawberry as BC-producing strain. In spite of *Glucanobacter xylinus* was the most efficient BC producer, but a newly strain in this study can be reach to maximum yield after optimization study. Therefore, the next experiment will be focused in the bio-valorization of different agriculture waste as a culture medium for highest BC yield via Response Surface Methodology (RSM).

**Fig. 3:**
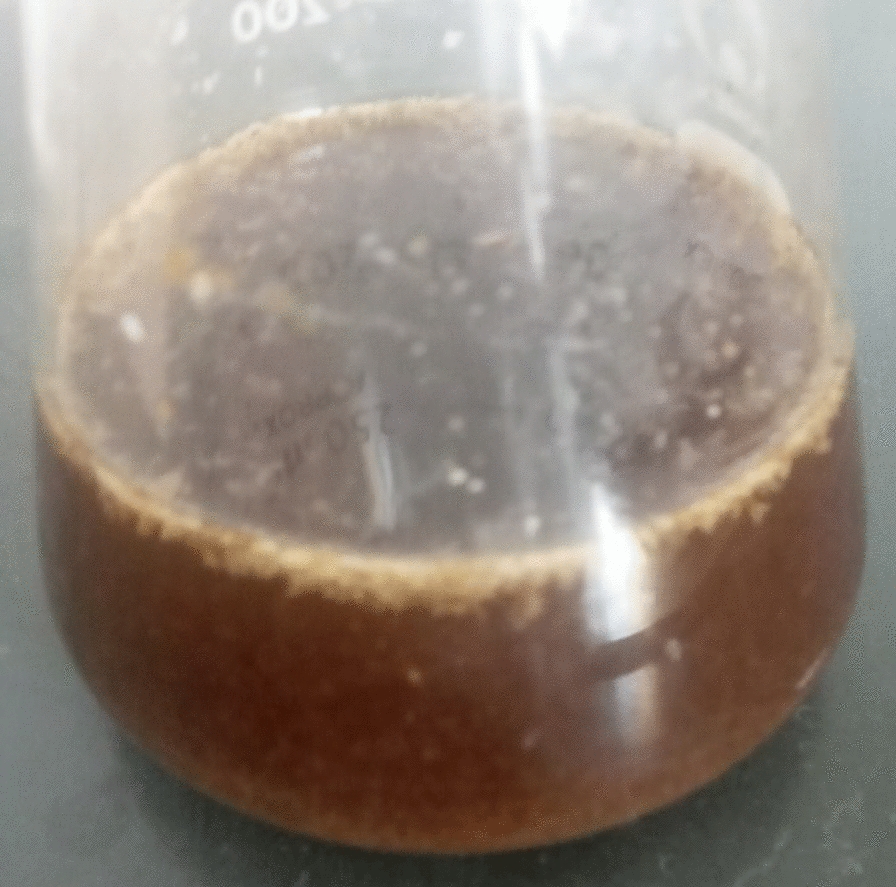
BC production by MPW

The urgent need for the BC is increased continuously, but the high cost of the production medium and low productivity is the most hurdle reasons in their commercialization. In this regard, there are countless of agricultural wastes could open the green ecofriendly way to valorized it to sustainable production of BC. In this respect, green utilization of different fruit peels by prepared it in the hydrolysate form to display as culture medium for the BC production by *Achromobacter* S33 was carried out. As shown in Table 3, BC yield was found so varied according to the hydrolysate used. Since, the maximum BC pellicles yield was found using MPW with 0.52 ± 0.04 g/L as shown in Fig. 3, followed by Pineapple Peel Waste (PPW) with 0.38 ± 0.05 g/L. Indeed, the BC productivity using MPW was greater than by using HS control medium (0.48 ± 0.02 g/L), with 0.04 g/L. Otherwise, the pomegranate peel waste, and citrus orange peel was correlated with significant inhibition of the BC productivity. Therefore, MPW culture medium was utilized for further optimization approach to improvement the BC production. Furthermore, the treatment of each of peel with nitric acid and justified the pH value of the medium with sodium hydroxide could be forming the sodium nitrate as resulting of the ion exchange between them, which play an important role in the growth of bacterial cells [1]. Surprisingly, the exploitation of MPW for BC production by a newly isolate *Achromobacter* S33 contributes in the reducing of environmental contamination, help in waste management and decrease agricultural residue disposal costs [7]. Our previous study was successfully exploitation the potato peel waste as a sustainable culture medium for the BC production by *Glucanobacter xylinum* [1].

**Table 3:** Screening of different fruit peels hydrolysate

Fruit peels	BC Productivity (g/L)
Apple	0.27 ± 0.05
Citrus orange	0.018 ± 0.00
Banana	0.32 ± 0.08
Pomegranate	0.006 ± 0.00
Pineapple	0.38 ± 0.05
Mango	0.52 ± 0.04
Standard HS culture medium	0.48 ± 0.02

### Optimization of BC production using CCD

Central composite and Box–Behnken designs are wildly used to make more interactions between factors and their levels for getting maximum of the target product. Therefore, CCD was used for enhancing bacterial cellulose production by *Achromobacter sp*.as shown in Table 4. Result illustrated that, run no. 10 was the best for bacterial cellulose production, and where was 1.23 g/l at conditions pH 5.0, inoculum size 6% and mango peel 40%. On the other hand, the lowest production appeared in run 14 with these conditions pH 3.0, inoculum size 4% and mango peel 40%. ANOVA results showed that, the model is highly significant where P value was 0.010 (Table 5). Moreover, pH was significant for bacterial cellulose production where P-value was 0.00. Likewise, square of pH only was significant for bacterial cellulose production where P-value was 0.002. On the other hand, both inoculum size and mango peel concentration were non-significant where P-value was 0.171 and 0.123 respectively. Likewise, two way interactions between each two were non-significant. Multiple regression analysis of the experimental data gave the second order polynomial equation for BC production in terms of uncoded factors is shown in regression equation as follow:

$${\text{BC }} = - {4}.{93} + {1}.{462} {\text{pH}} + 0.{4}0{6} {\text{IS}} + 0.0{489} {\text{MP}} - 0.{1116} {\text{pH}}*{\text{pH}} - 0.0{275} {\text{IS}}*{\text{IS}}$$

$$- \,0.000{26}0 {\text{MP}}*{\text{MP}} - 0.0{12}0 {\text{PH}}*{\text{IS}} - 0.00{297} {\text{PH}}*{\text{MP}} - 0.00{2}0{3} {\text{IS}}*{\text{ MP}}$$

**Table 4:** BC production using CCD design

Run Order	pH	Inoculum size	MP %	BC productivity	Predicted
1	5	4	40	1.007	1.103375
2	4	5	30	0.711	0.755438
3	6	3	30	1.15	1.041688
4	6	5	50	1.06	1.151938
5	4	3	50	0.822	0.804188
6	5	4	40	1.21	1.103375
7	5	4	20	0.945	1.011271
8	5	4	60	1.29	1.218271
9	5	2	40	0.89	1.018521
10	5	6	40	1.233	1.199021
11	5	4	40	1.2	1.218688
12	7	4	40	1.111	1.129771
13	5	4	40	1.221	1.218688
14	3	4	40	0.439	0.414771
15	6	5	30	1.16	1.183271
16	5	4	40	1.099	1.138208
17	4	5	50	0.799	0.912771
18	4	3	30	0.722	0.635521
19	6	3	50	1.2	1.161021
20	5	4	40	1.189	1.138208

**Table 5:** Analysis of Variance (ANOVA)

Source	DF	SS	MS	P-Value
Model	11	0.909	0.082	0.010
Linear	3	0.586	0.195	0.002
pH	1	0.511	0.511	0.000
IS	1	0.032	0.032	0.171
MP	1	0.042	0.042	0.123
Square	3	0.299	0.099	0.013
pH*pH	1	0.298	0.298	0.002
IS*IS	1	0.018	0.018	0.294
MP*MP	1	0.016	0.016	0.320
2-Way interaction	3	0.011	0.003	0.848
pH*IS	1	0.001	0.001	0.784
pH*MP	1	0.007	0.007	0.503
IS*MP	1	0.003	0.003	0.646
Error	8	0.115	0.014	
Lack of fit	5	0.090	0.018	0.278
Pure error	3	0.024	0.008	
Total	19	1.024		

**Fig. 4:**
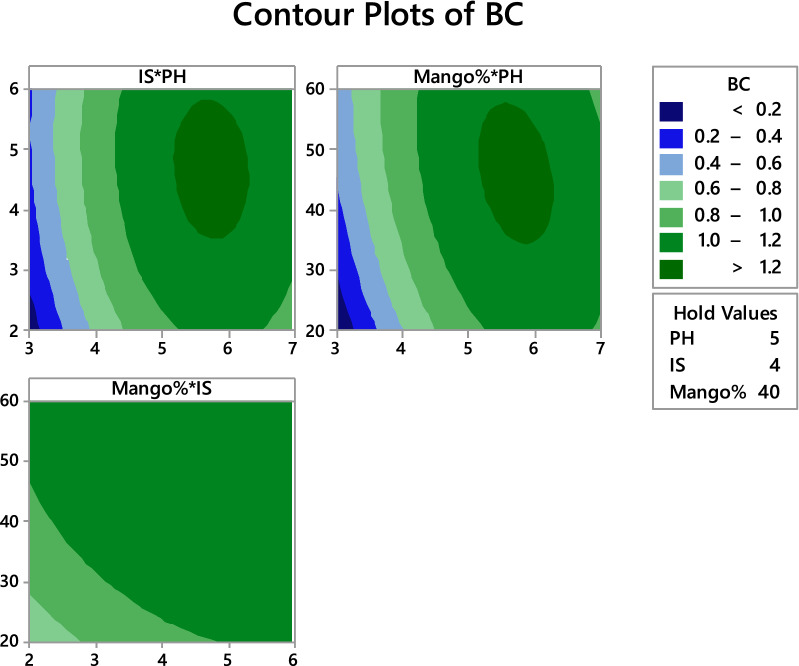
Contour plots of factors affecting BC production

**Fig. 5:**
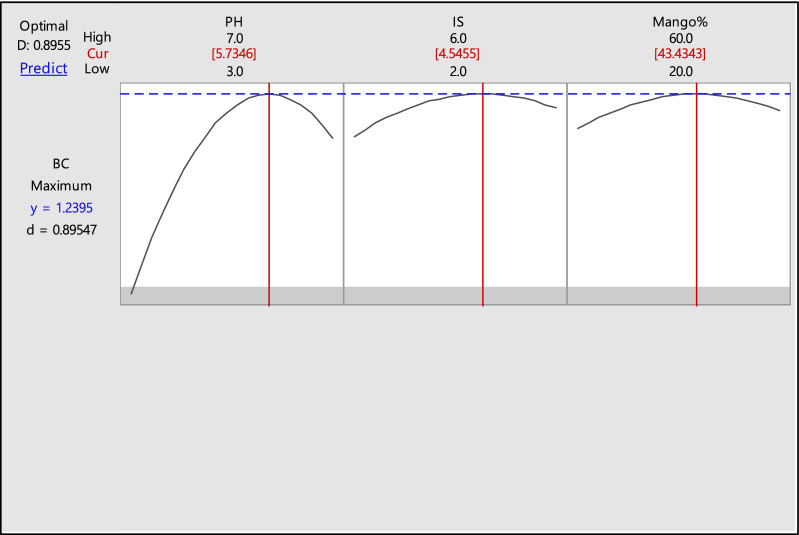
Response optimizer for BC production by *Achromobacter sp*

To study the interactive effect of two factors on the BC production, the response surface methodology was used contour plot was designed. Each plot measure the function of two factors at a time, maintaining all other factors at zero levels, this helps in understanding the interaction between these two factors. A circular contour plot implies that interactions are insignificant between the corresponding variables, while an elliptical contour plot suggests that the interactions between the selected variables are significant. Contour plots of different factors are presented graphically in Fig. 4. The interaction between mango peel percentage and pH of the medium showed that the maximum production of BC could be obtained when pH 5.5–6.0 with 40–50% mango peel. In the interaction between inoculum size and pH of the medium, the maximum production of BC at inoculum size 4–5% and pH at 5.5–6.0. Consequently, these data confirmed that pH is the most factor affected BC production. Also, Fig. 5 shows pH was the significant for BC production among other factors, meanwhile all interactions between each two factors (pH, inoculum size and mango peel) are non-significant, also this confirms by ANOVA results.

To validate our results, response optimizer was performed which predict the yield at definite condition. In this study, the model predicted the produced bacterial cellulose 1.239 g/L theoretically at conditions pH 5.73, inoculum size 4.54% and mango peel 43.4%. Practically, *Achromobacter sp.* produced bacterial cellulose 1.262 ± 0.07 g/L when is grown on optimized conditions, where this is higher than polynomial equation predicted value, demonstrating the accuracy of the statistical model employed to optimize bacterial cellulose production.

Different fruit peels are used for bacterial cellulose production from bacteria [33]. Güzel and Akpınar [33] reported that kiwi fruit peel hydrolysate was the higher for bacterial cellulose by *Komagataeibacter hansenii* GA2016 among other fruit peels. Padmanabhan, Lionetto, Nisi, Stoppa and Licciulli [34] produced crystalline bacterial cellulose by *Gluconacetobacter xylinus* using orange peel extract as a substrate. Likewise, orange peel extract was used for bacterial cellulose production by *K. sucrofermentans* DSM 15973. Another study, pine apple and watermelon peels were used for bacterial cellulose production by *K. hansenii* under static conditions [35]. In this study, bacterial cellulose was produced by *Achromobacter sp.* using mango peel waste (43.4%) as a substrate for the first time. The pH scale measures hydrogen ion (H^+^) concentration, which affects enzyme activity, thus influencing the bacterial growth. High pH corresponds to low concentration of H + , while low pH corresponds to high concentration of H^+^. A neutral pH is when H^+^ and OH − (hydroxyl ions) are equal [36]. In the current study, pH factor was the highest for BC production by *Achromobacter sp.* using mango peel waste among other used factors, where pH at 5.73 was the highest for BC production. Hwang, Yang, Hwang, Pyun and Kim [37] reported that, pH at 5.5 was the best for BC production using *Acetobacter xylinum* BRC5. Zahan, Pa’e and Muhamad [38] studied the effect of pH on bacterial cellulose production by *Acetobacter xylinum* 0416 and found the highest yield was obtained at pH 5.0. Aswini, Gopal and Uthandi [39] optimized the BC production by *Acetobacter senegalensis* MA1 using RSM-CCD design, and found the highest production at pH 6.0*.* Suwanposri, Yukphan, Yamada and Ochaikul [40] illustrated that, pH at 6.21 was the highest for BC production by *Komagataeibacter sp* PAP1 using soya bean whey. On the other hand, other studied confirmed that pH of the medium between 3.5 and 4.5 is the best for BC production by *Gluconacetobacter medellensis* [41]*, Gluconacetobacter* sp. gel_SEA623-2 [42] and *G. sucrofermentans* B-11267 [43].

### BC Characterization

**Fig. 6:**
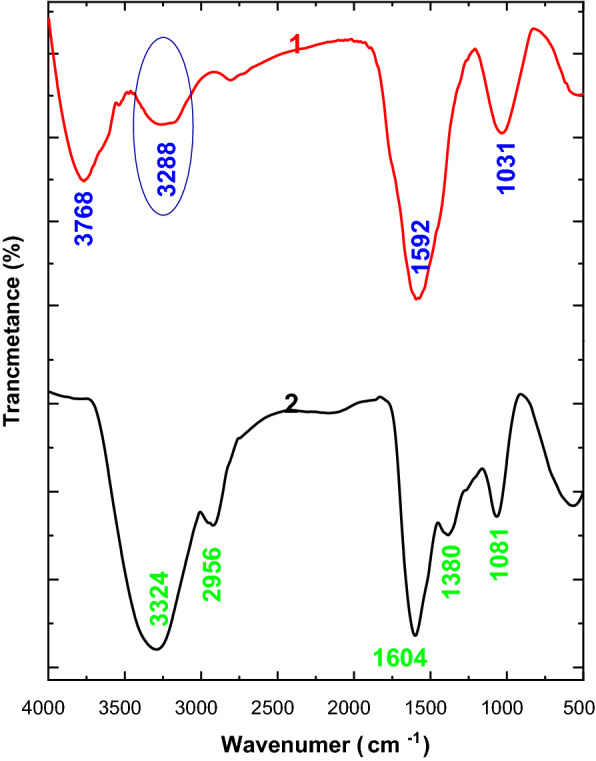
AT-FTIR spectra of produced nanofibers BCs using HS (1) and MPW (2) media

The physicochemical study of the BC nanofibers which produced using HS and MPW media includes ATR-FTIR and XRD (Fig. 6). The sample 1 represented the typical absorbance of BC at 3768, 3288 (broad band), 1592 and 1031 cm^−1^ corresponding to O–H stretching vibration, CH stretching frequency, band, HCH & OCH bending inside of plane vibration and carbohydrate glycosidic bond characteristic band [23, 44]. On the other hand, the sample of MPW was observed a characteristic bands at 3324 cm^−1^ that referred to O–H stretching vibration that is response as a significant shifting to lower frequency in compassion with HS spectrum this in a nice agreement with the previous work [44, 45]. Likewise, the CH stretching band was completely changed in MPW spectrum where the band was observed as a small band with low intensity at 2956 cm^−1^. This observation is referred to hydrogen bond strength that is stronger in nanoform of cellulose. In addition, the band at 1604 cm-1 that reffered to CH2 symmetric stretching intensity was reduced as well as band at 1380 cm-1 of CH3 and OH deformation was assigned that were due to high crystallinity of MPW than HS BC [46, 47]. In addition to, the carbohydrate band at 1081 cm^−1^ that referred to BC active groups with a significant shift in bands position and intensity [1, 7]. These results may be related to the change in the intermolecular structure of the BC produced from the both medium due to the main chemical component of production media. Over all, the above results affirmed the samples 1 and 2 are a cellulose with different inter and intra molecular structure that could be refereed to samples crystallinity.

**Fig. 7:**
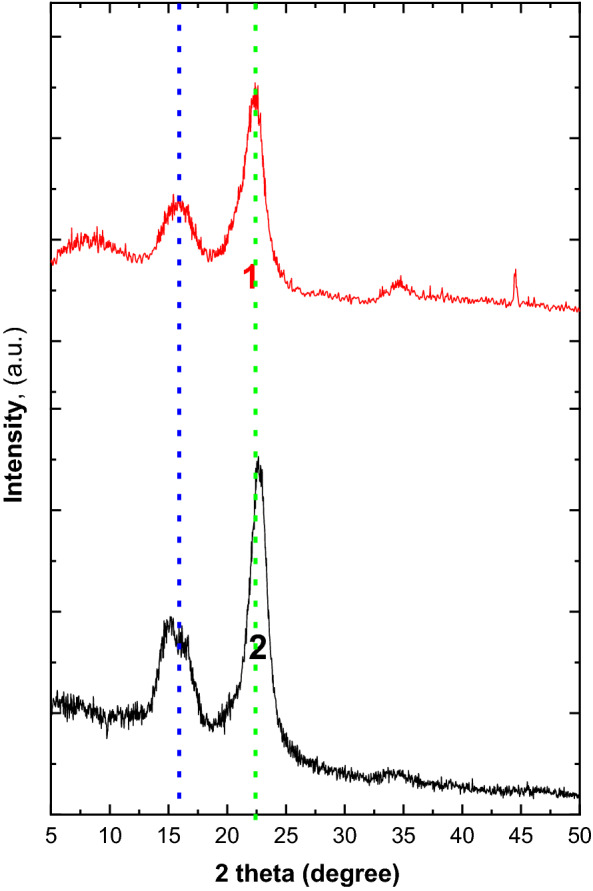
XRD pattern of produced nanofibers BCs using HS (1) and MPW (2) media

Additionally, the X-ray diffraction crystallography of BCs confirmed appearance of two oriented main characteristic peaks around 15° and 22° as shown in Fig. 7 that closely with the pure BC XRD pattern as reported in many literatures [1, 7]. In this context, both BCs were appeared as a typical structure of cellulose with different crystallinity that as 72 and 79% for BC produced form HS and MPW, respectively.

**Fig. 8:**
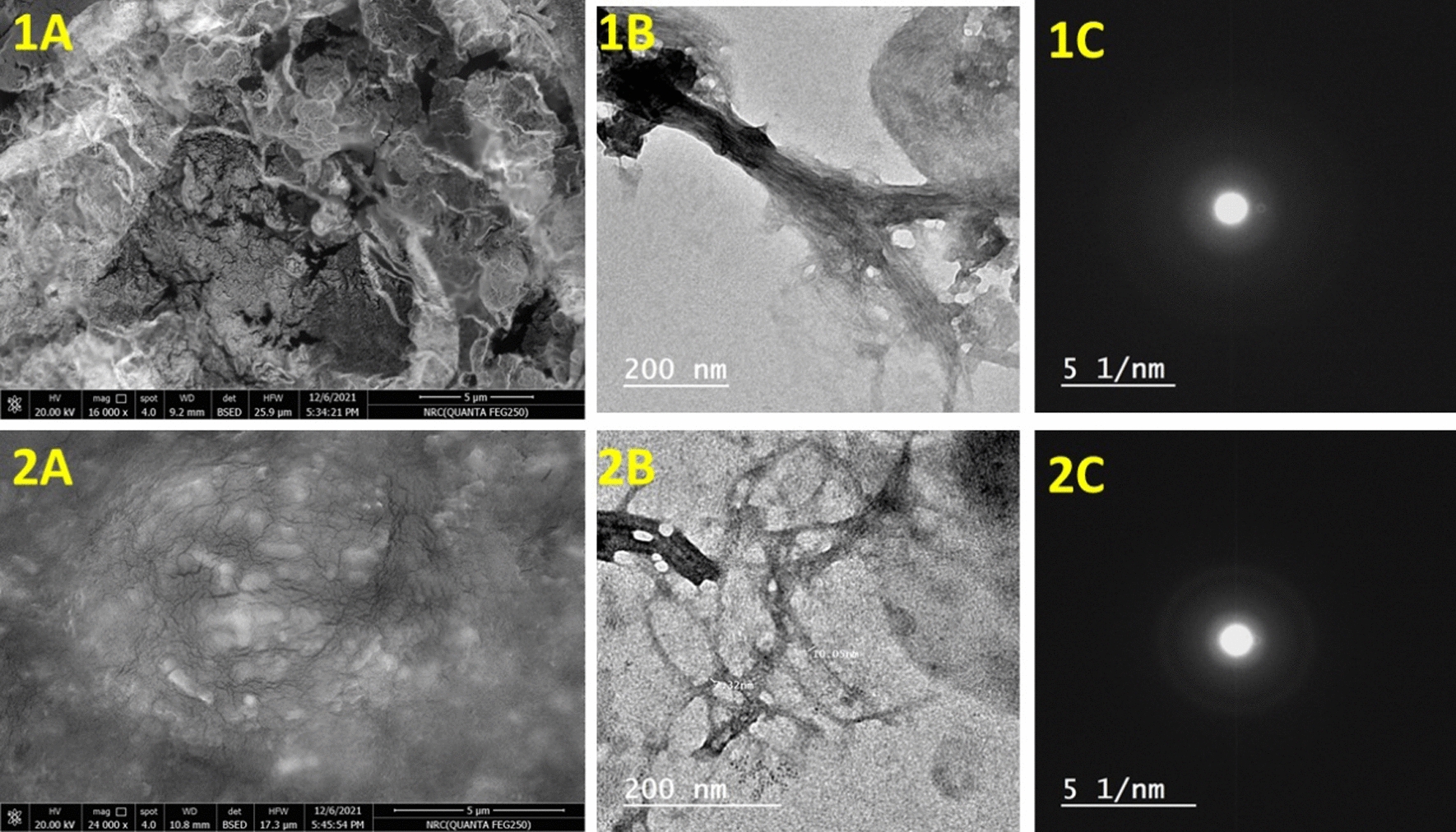
SEM image of nanofibers BCs using HS (1**A**) and MPW (2**A**) media. The TEM image of nanofibers BCs using HS (1**B**) and MPW (2**B**) media. SAED pattern of nanofibers BCs using HS (1**C**) and MPW (2**C**) media

Figure 8 shows SEM as well as TEM with SAED pattern. Results illustrated that, SEM image of Sample 1 (Fig. 8-1A) appeared with a rough surface related to fibrous structure of cellulose. Additionally, the TEM image (Fig. 8-1B) was presented as a nanofibrous structure collected as wisp. The SAED pattern (Fig. 8-1C) appeared as circuits fused together. On the other hand, the sample 2 SEM image (Fig. 8-2A) was observed as a fibrous structure appearance with lower condensing in comparative with sample 1. Additionally, the TEM image (Fig. 8-2B) confirmed that the fibers was observed with little condensing in comparison with the sample 2. Moreover, the SAED pattern was appeared as separated circuits that referred to the higher crystallinity (Fig. 8-2C). These results were in agreement with the XRD crystallographic study conclusion. In addition to, nanofibers dimensions diameter and length as about 10 and 200 nm, respectively.

## Conclusion

The *Achromobacter* S3 strain was selected from eight cellulose producer isolates that represented a high BC productivity using zero-value waste medium (MPW). Furthermore, the physiochemical and topographical characterizations studies are emphasized that the produced BC from *Achromobacter* S3 strain using both media (HS and MPW) are performed as a typical cellulose structure. Moreover, the medium type was affect the crystallinity of produced BCs as well as the intermolecular structure of the cellulosic fibers and the cellulosic fibers dimensions were observed nonsignificant effect in both media. The HS medium produced low crystallinity fibers with wide swipes collection as well as MPW medium was produced high crystallinity fibers that affirmed the produced BC is nanofibers.

## Data Availability

The data and materials were mentioned in the manuscript and the data available upon requested.
